# Coronary endothelial cells receive Notch1 signal during heart development

**DOI:** 10.1016/j.bbrep.2026.102744

**Published:** 2026-08-07

**Authors:** Yoshitoku Watabe, Satoru Takahashi, Masaharu Yoshihara

**Affiliations:** a College of Medicine, School of Medicine and Health Sciences, University of Tsukuba, 1-1-1, Tennodai, Tsukuba, Ibaraki, 305-8575, Japan; b Department of Anatomy and Embryology, Institute of Medicine, University of Tsukuba, 1-1-1, Tennodai, Tsukuba, Ibaraki, 305-8575, Japan; c Laboratory Animal Resource Center, Transborder Medical Research Center, Institute of Medicine, University of Tsukuba, 1-1-1, Tennodai, Tsukuba, Ibaraki, 305-8575, Japan

**Keywords:** Cre/loxP system, Gal4/UAS system, Embryo, Endocardial cells, Single-cell RNA-Sequencing, Transgenic animal

## Abstract

The Notch1 signaling pathway, via the Notch1 receptor and its endogenous ligands, has been suggested to play a role in heart development, as mutations in Notch1 and associated genes are associated with abnormal development of the myocardium, aortic valve, and outflow tract. The cell lineages receiving this signaling are, however, still unclear, partly owing to technical difficulties in labeling Notch1 signaling rather than Notch1 receptor expression. To identify the cell lineages that receive Notch1 signal during heart development, we re-analyzed publicly available single-cell RNA-sequencing data from human embryos (post-conception weeks 6, 8, 12, and 19) and mouse embryos (embryonic days 12.5, 14.5, and 16.5). We found that endothelial cells expressed Notch1 in both species. To confirm Notch1 signaling rather than Notch1 receptor expression in heart development, we carried out transgenic mouse experiments that enabled tracing of both past and ongoing Notch1 signaling by combining the Notch1 receptor protein with the Gal4/UAS and Cre/loxP systems. Past Notch1 signal analysis confirmed that coronary endothelial cells, in addition to a small proportion of endocardial cells, received Notch1 signals during development, consistent with our findings in single-cell RNA-seq re-analysis. Ongoing Notch1 signaling was observed in the surface-covering cells of the vascular lumens, although its colocalization with an endothelial marker (Pecam1/Cd31) was unclear. Collectively, our findings suggested that coronary endothelial cells are Notch1 signal receivers, which should contribute to understanding the pathogenesis of congenital heart diseases.

## Highlights

• Single-cell analysis revealed Notch1 expression in coronary endothelial cells.
• Coronary endothelial cells were labeled with past Notch1 signal in adult mice.
• Luminal surface cells in the mouse embryonic heart showed ongoing Notch1 signal.

## 1 Introduction

Notch signaling is a cell-cell communication signaling in which four receptors (Notch1, Notch2, Notch3, Notch4) release their intracellular domain upon binding to their ligands on the adjacent cell membrane [1]. The released intracellular domain of Notch receptors induces expression of many downstream genes, including Hes/Hey family genes and tissue-specific genes. Importantly, there is a clear difference between Notch receptor expression and Notch signaling, since the latter requires binding of the Notch receptor to its endogenous ligands between cells.

Notch signaling is associated with the development of many organs, including the brain, heart, digestive tract, and kidney, suggesting that this signaling is associated with a wide variety of cells across all three germ layers. In addition to its role in parenchymal cell development, Notch signaling is involved in stromal development, such as sprouting angiogenesis [2], underscoring its complex role in many organ developments.

Involvement of Notch signaling in heart development is suggested by human patients with genetic mutations in Notch signaling components. For example, four mutations (p.R1108X) (p.H1505del) [3] (p.A1343V and p.P1390T) [4] in the extracellular domain of NOTCH1 have been associated with bicuspid aortic valve (BAV). In addition, four mutations in the extracellular domain (p.G661S, p.A683T, p.E694K, p.R1608H) and one mutation in the intracellular domain (p.V2536I) of NOTCH1 were associated with left ventricular outflow tract malformations with some overlap with BAV [5]. Furthermore, two mutations (p.R530X) (p.V943F) in MIB1 that promote endocytosis of Notch ligands were associated with left ventricular noncompaction cardiomyopathy (LVNC) [6]. These reports suggest involvement of Notch signaling in heart development at least for NOTCH1.

Other Notch receptors also act in heart development. Notch2 is associated with cardiac defects via its loss-of-function mutations in Alagille syndrome (c.5930–1G→A) (c.1331G→A) [7] (c.4819C→T) [8] (p.Y222C) (p.V1623G) (p.D2004V) (p.R2003*) [9] and via its gain-of-function mutation in Hajdu–Cheney syndrome (p.E2143X) [10]. The gain-of-function mutation in Hajdu–Cheney syndrome (p.E2143X) has been suggested to act in a cell-autonomous manner in the myocardium, as overexpression of the human NOTCH2 intracellular domain in mouse cardiomyocytes suppressed purine nucleotide metabolism and led to subsequent cardiac hypertrophy [11]. Another Notch gene, Notch3, is associated with a vascular disease, CADASIL, and is suggested to act in vascular smooth muscle cells of the heart but not in cardiomyocytes [12]. In summary, Notch2 and Notch3 have already been linked to cardiomyocytes and vascular smooth muscle cells, respectively, in the developing heart. To the best of our knowledge, Notch4 has not been linked to normal heart development. In contrast to Notch2 and Notch3, Notch1 has not been systematically examined for its role in cell identity, as we will discuss later.

Heart development is a complex process in which many cell lineages interplay. In the evolution from fish to mammals, the number of chambers and the thickness of the walls increased, requiring additional structures such as compact ventricular walls [13] and coronary vessels to support thick myocardium [14], suggesting that evolution increased the diversity of cell types in the heart. In addition, cellular heterogeneity has been suggested in endothelial cells, for example, since Fabp4 and Nfatc1 mark the coronary or sinus venosus endocardial origins, respectively [15]. Therefore, to understand the cellular interplay during heart development, gene expression profiles should be described at high resolution.

Notch1 was expressed in endocardial progenitor cells rather than cardiomyocyte progenitor cells among cardiac progenitor cells with Mesp1 expression around embryonic days (E) E6.5 and E7.5, although Notch1-tracing using Notch1-CreERT2 mice at this stage suggested that small proportions of Notch1-expressing cells could also contribute to epicardial cells and cardiomyocytes [16]. Indeed, the Notch1 intracellular domain was detected in several regions, including the E8.5 ventricular endocardium and the E9.5 endocardium of the atrioventricular canal and outflow tract [17]. Importantly, Tnnt2-Cre-mediated cardiomyocyte-specific deletion of Notch1 and common Notch effector Rbpj had no apparent heart abnormalities, while Rbpj deletion in myocardial and endocardial progenitor cells via Nkx2.5-Cre resulted in ventricular septal defect (VSD), double outlet right ventricle (DORV), and BAV [18], underpinning still unclarified Notch signaling action via several receptors or in multiple cell types. Although Notch signaling in heart development has already been extensively reviewed [19], cell lineage tracing that can overcome the cleavage-dependent nature of the Notch1 receptor and the high cellular heterogeneity in the heart should contribute to understanding heart development.

In this study, we combined single-cell RNA-sequencing (scRNAseq) re-analyses and a transgenic reporter assay that could label both past and ongoing Notch1 signaling rather than Notch1 receptor expression to examine the cell lineages that receive the Notch1 signal.

## 2 Materials and methods

### 2.1 Single-cell re-analysis of the human embryo

We used processed scRNAseq data (https://doi.org/10.5281/zenodo.7063223) [20] containing human embryonic heart tissues at post-conception week 6 (PCW6), PCW8, PCW12, and PCW19, which were originally reported by Suryawanshi et al. [21], Asp et al. [22], Miao et al. [23], and Cui et al. [24]. These data contained two samples named “PCW6” and “PCW6_ASP ET AL” for the time point PCW6. We used essentially the same annotation strategy as the original report by Ameen et al. [20]. Cell numbers in each subset of each sample, and percent expression and average expression of genes in the dot plots are available at Supplementary Materials 1 and 2, respectively. In addition, the top 5 and all differentially expressed genes (DEGs) for each cell subset are available in Supplementary Materials 3 and 4.

### 2.2 Single-cell re-analysis of mouse embryo

We used scRNAseq data from mouse embryonic hearts at E12.5, E14.5, and E16.5, for each of which replicates were prepared (Gene Expression Omnibus Accession Number: GSE230531) [25]. We excluded scRNAseq data from E8.5 and E10.5 that were also provided by Ren et al. [25] since these data included trunk region cells as well as cardiac cells. Single-cell re-analyses of both human and mouse data were conducted in the Seurat pipeline [26]. The quality control parameters are available in Supplementary Material 5, and all R code used in this study is archived on Zenodo (see the “Data availability statement”). In addition, cell numbers in each subset of each sample, and the percent and average gene expression in the dot plots for mouse data, are also available in Supplementary Materials 6 and 7, respectively. Also, the top 5 and all DEGs for all cell subsets are available in Supplementary Materials 8 and 9.

### 2.3 Animal experiments

Animal experiments were conducted in accordance with institutional and national guidelines. Approval was obtained from the Institutional Animal Care and Use Committee and the DNA Experiment Committee of the University of Tsukuba (Approval Numbers for Animal Experiments: 22–059, 23-049, 24-133, 25-150, 26-305) (Approval Number for DNA Experiments: 220018).

We used three transgenic mouse lines: Notch1-Gal4VP16 [27], UAS-Cre [28], and R26GRR [29]. Briefly, Notch1-Gal4P16 mouse expresses Gal4VP16-fused Notch1 receptor under the endogenous Notch1 promoter, which allows free Gal4VP16 when this fusion receptor binds to endogenous Notch1 ligands. Free Gal4VP16 binds to the UAS sequence in the UAS-Cre mouse to induce Cre recombinase expression. Then, Cre recombinase excises the EGFP sequence and alternatively induces tandem DsRed (tDsRed) under the CAG promoter. Using these three transgenic mouse lines enables precise labeling of both ongoing and past Notch1 signaling, which is difficult to achieve with labeling of endogenous genes [30].

Past Notch1 signal analysis in adult mouse samples by immunohistochemistry (Fig. 3) was repeated three times (N = 4 for Notch1-Gal4VP16; UAS-Cre; R26GRR mice and N = 1 for UAS-Cre; R26GRR mouse as a negative control). Ongoing Notch1 signal analysis at E14.5 by immunohistochemistry (Fig. 4) was repeated three times (N = 3 for Notch1-Gal4VP16; UAS-Cre embryos and N = 1 for UAS-Cre embryos as a negative control). Ongoing Notch1 signal was also examined at the adult stage using a Notch1-Gal4VP16; UAS-Cre; R26GRR mouse (Supplementary Material 15). For all immunohistochemistry experiments, we performed antigen retrieval using DAKO Target Retrieval Solution (Cat #: S1699, Agilent Technologies, Santa Clara, CA, USA) generally at 105° for 5 min (121° for 5 min for one sample with the anti-Cre antibody). Then, the paraffin sections were sequentially incubated with a rabbit polyclonal anti-RFP antibody (Cat #: 600-401-379, Lot #: 46317, RRID: AB_2209751, Rockland Immunochemicals, Philadelphia, PA, USA) (1:200 or 1:250 dilution, 1 h, room temperature) or a rabbit monoclonal anti-Cre antibody (clone: D7L7L, Cat #: 15036S, RRID: AB_2798694, Cell Signaling Technology, Danvers, MA, USA) (1:200 dilution, 1.5 h, room temperature), and Histofine SimpleStain (Cat #: 414341, Nichirei Biosciences, Tokyo, Japan), Histofine DAB Substrate Kit (Cat #: 425011, Nichirei Biosciences) and counterstained with Mayer's hematoxylin solution (Cat #: 131–09665, Fujifilm, Osaka, Japan). Images were captured using BIOREVO-BZ-X810 (Keyence, Osaka, Japan).

For immunofluorescence for EGFP, tDsRed, and Pecam1/Cd31 (Supplementary Material 14), we used Notch1-Gal4VP16; UAS-Cre; R26GRR adult mouse hearts (N = 4) and performed antigen retrieval using DAKO Target Retrieval Solution (Cat #: S1699, Agilent Technologies) at 105° for 5 min. The paraffin sections were sequentially incubated with chicken anti-GFP antibody (Cat #: GFP-1020, Lot #: GFP3717982, RRID: AB_10000240, Aves Labs, Davis, CA, USA, 1:2000 dilution), rabbit anti-RFP antibody (Cat #: 600-401-379, Lot #: 46317, RRID: AB_2209751, 1:250 dilution) and rat anti-Pecam1 (Cd31) antibody (Cat #: 14-0311-81, Lot #: 3306657, RRID: AB_467200, eBioscience, San Diego, CA, USA, 1:100 dilution) at 4° overnight, and then, with Alexa Fluor Plus 488-conjugated goat anti-chicken IgY antibody (Cat #: A32831, Lot #: XB343360, RRID: AB_2762843, Thermo Fisher Scientific, Waltham, MA, USA, 1:1000 dilution), Alexa Fluor Plus 555-conjugated goat anti-rabbit IgG antibody (Cat #: A32732, Lot #: XF348170, RRID: AB_2633281, Thermo Fisher Scientific, 1:1000 dilution) and Alexa Fluor 647-conjugated donkey anti-rat IgG antibody (Cat #: ab150155, Lot #: 1107794-6, RRID: AB_2813835, Abcam, Cambridge, UK, 1:1000 dilution) at room temperature for 1 h, and finally with Hoechst33342 (final concentration: 1 μg/mL) at room temperature for 5 min. Immunofluorescence for Pecam1/Cd31 and Cre was performed using three embryonic day 14.5 Notch1-Gal4VP16; UAS-Cre mouse embryos (Supplementary Material 16). We performed antigen retrieval using DAKO Target Retrieval Solution (Cat #: S1699, Agilent Technologies) at 105° for 5 min. Then, we incubated the samples with a rabbit monoclonal anti-Cre antibody (clone: D7L7L, Cat #: 15036S, RRID: AB_2798694, Cell Signaling Technology, 1:200 dilution) and rat anti-Pecam1 (Cd31) antibody (Cat #: 14-0311-81, Lot #: 3306657, RRID: AB_467200, eBioscience, 1:100 dilution) for 1.5 h at room temperature. Finally, we incubated the samples with Alexa Fluor Plus 488-conjugated goat anti-rabbit IgG antibody (Cat #: A32731, Lot #: XH353659, RRID: AB_2633280, Thermo Fisher Scientific, 1:1000 dilution) and Alexa Fluor 647-conjugated donkey anti-rat IgG antibody (Cat #: ab150155, Lot #: 1107794-6, RRID: AB_2813835, Abcam, 1:1000 dilution) at room temperature for 1 h, and with Hoechst33342 (final concentration: 1 μg/mL) at room temperature for 5 min. Images were captured using BIOREVO-BZ-X810, and their brightness, but not contrast, was adjusted in Adobe Photoshop.

## 3 Results

### 3.1 Single-cell re-analysis of the human embryo

Since Notch signaling is involved in multiple cardiac abnormalities in humans, we started by screening scRNAseq data of human embryonic hearts. The original paper [20] provides an integrated and quality-controlled RDS file, and UMAP analysis revealed overlap among the five samples (PCW 6, 8, 12, 19, and PCW6_ASP ET AL) (Fig. 1A). These samples contained endothelial cells, including arterial, capillary, venous, and lymphatic endothelial cells (aEC, Cap, vEC, lEC, respectively) and endocardial cells (Endo1), revealed by CDH5 (pan-endothelial marker) and CDH11 (endocardial marker) expressions and marker gene analysis (Fig. 1B, C, 1D). We examined expression of Notch genes (NOTCH1, NOTCH2, NOTCH3, NOTCH4) and Notch downstream target genes (HES1, HES2, HES3, HES4, HES5, HES6, HES7, HEY1, HEY2, HEYL) and found that NOTCH1 was expressed primarily in endothelial cells including endocardial cells although NOTCH1 was also expressed in other stromal cells such as smooth muscle cells (SMC) and pericytes (PC) annotated as same as the original report [20] (Fig. 1E and F). To further clarify their statistical significance, we examined enrichments of NOTCH1 expression in each cell subset by the Wilcoxon Rank Sum test in the “FindAllMarkers” function of the Seurat package and found that endothelial cells (aEC, Cap, lEC), endocardial cells (Endo1), and pericytes (PC) significantly expressed NOTCH1 (Supplementary Material 10). NOTCH2 and NOTCH3 were expressed in PC and SMC, while NOTCH4 was expressed in aEC and Cap (Fig. 1E). We consider that this caused HES1 and HES4 expressions across the cell types (Fig. 1E). Collectively, re-analysis of human embryonic hearts suggested that NOTCH1 was closely associated with, but not limited to, endothelial cells.

**Fig. 1:**
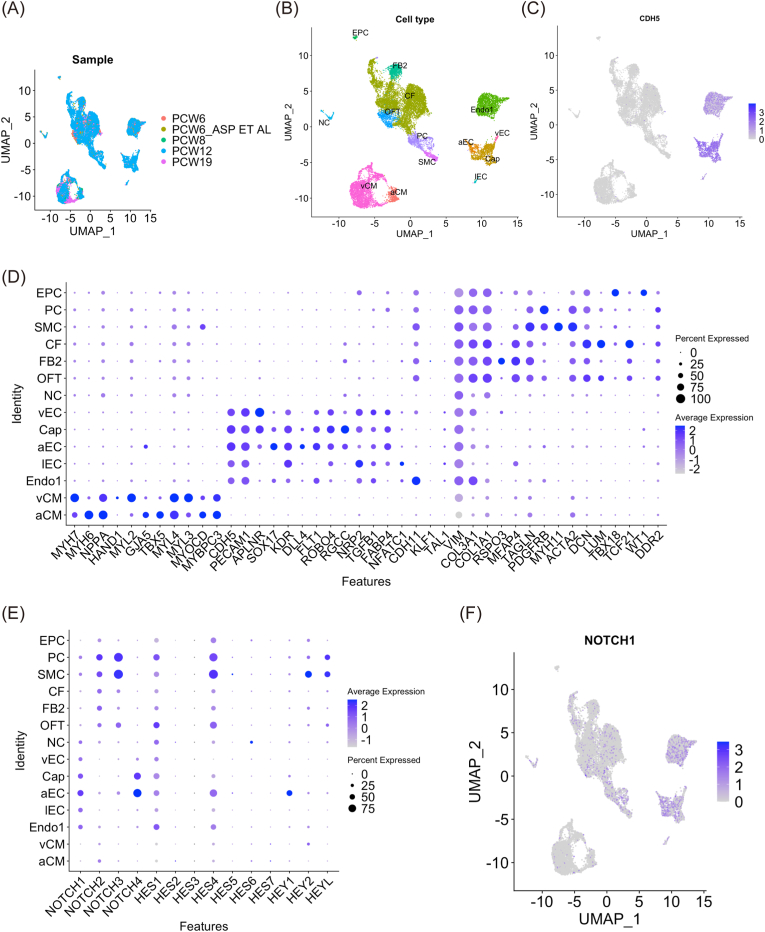
Re-analysis of scRNAseq data of human embryonic hearts (A) UMAP analysis of the four samples from PCW 6, 8, 12, 19, and PCW6_ASP ET AL. (B) UMAP analysis of annotated cell types. (C) CDH5 (pan-endothelial marker) expression in endothelial cells and endocardial cells. (D) Dot plot of marker genes. (E) Dot plot of Notch receptor genes and their downstream genes. (F) NOTCH1 expression in endothelial cells, endocardial cells, and other stromal cells such as pericytes.

### 3.2 Single-cell re-analysis of mouse embryo

To further characterize Notch1 expression in the developing heart, we also re-analyzed scRNAseq data from mouse embryos at E12.5, E14.5, and E16.5 [25]. In the UMAP analysis, we observed partial overlap among the six samples for which replicates were prepared (Fig. 2A). Then, we identified 24 cell subsets by using marker gene expressions (Fig. 2B and D). Pan-endothelial cell markers, Cdh5 (Fig. 2C) and Pecam1 (Fig. 2D), were expressed in Endothelial Cells 2-5. Although Endothelial Cell 1 lacked Cdh5 or Pecam1 expressions, this cell subset expressed another endothelial cell marker gene (Fabp5) and was located adjacent to Endothelial Cells 2 and 3, supporting the validity of the annotation. Larger-sized images of Fig. 2B and D are available in Supplementary Materials 11 and 12, respectively. Importantly, this scRNAseq data contained Cdh11-expressing endocardial cells (Endothelial Cell 2 subset) (Fig. 2D). Importantly, Notch1 and Hes1 expressions were observed in endothelial cells (Endothelial Cells 2-5), including putative endocardial cells (Endothelial Cell 2 subset) (Fig. 2D, E, and 2F). To further clarify their statistical significance, we examined enrichments of Notch1 expression in each cell subset by the Wilcoxon Rank Sum test in the “FindAllMarkers” function of the Seurat package and found that endothelial cells (Endothelial Cell 2-5), Epicardial Cell 1, and AVC 1 and 2 significantly expressed Notch1 (Supplementary Material 13). Since Hes1 has multiple upstream regulators, such as Smad and Sonic hedgehog signaling [31], we consider that Hes1 expression alone is not sufficient to conclude that Notch1 signaling is active. In addition, expression profiles of Fabp4 (a marker for endothelial cells of coronary origin) and Nfatc1 (a marker for endothelial cells of sinus venosus endocardial origin) [9] supported heterogeneity among endothelial cells in the heart, warranting histological examinations (Fig. 2G and H).

**Fig. 2:**
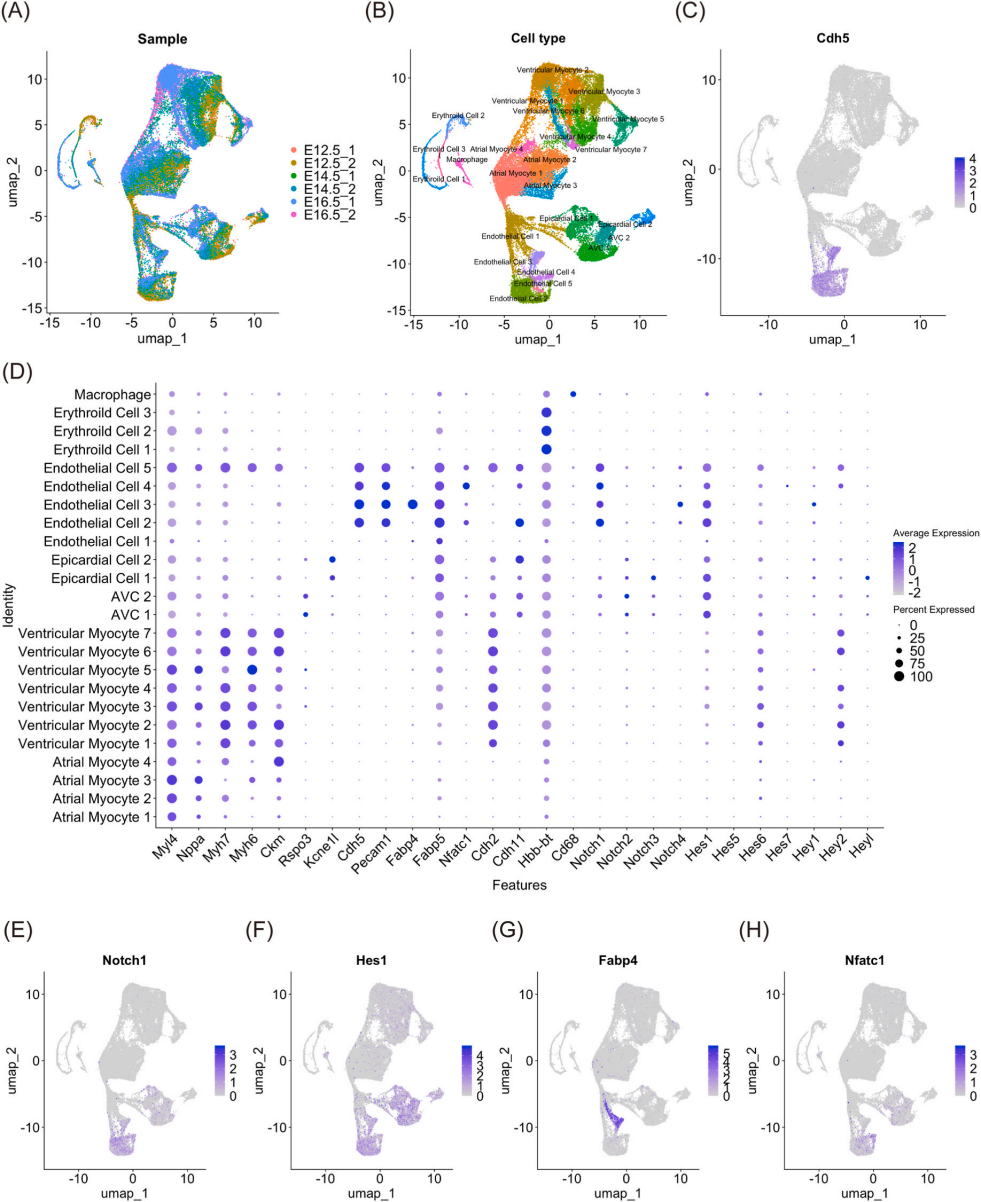
Re-analysis of scRNAseq data of mouse embryonic hearts (A) UMAP analysis of the seven samples after harmonization. (B) UMAP analysis of annotated cell types. (C) Cdh5 (pan-endothelial marker) identified endothelial cells, including endocardial cells. (D) Dot plot of marker genes. (E) Notch1 expression primarily in endothelial cells. (F) Hes1 expression in endothelial cells, epicardial cells, and atrioventricular canal cells. (G) Fabp4 expression marked a subset of endothelial cells (Endothelial Cell 4 subset). (H) Nfatc1 marked some endothelial cells differently from Fabp4.

**Fig. 3:**
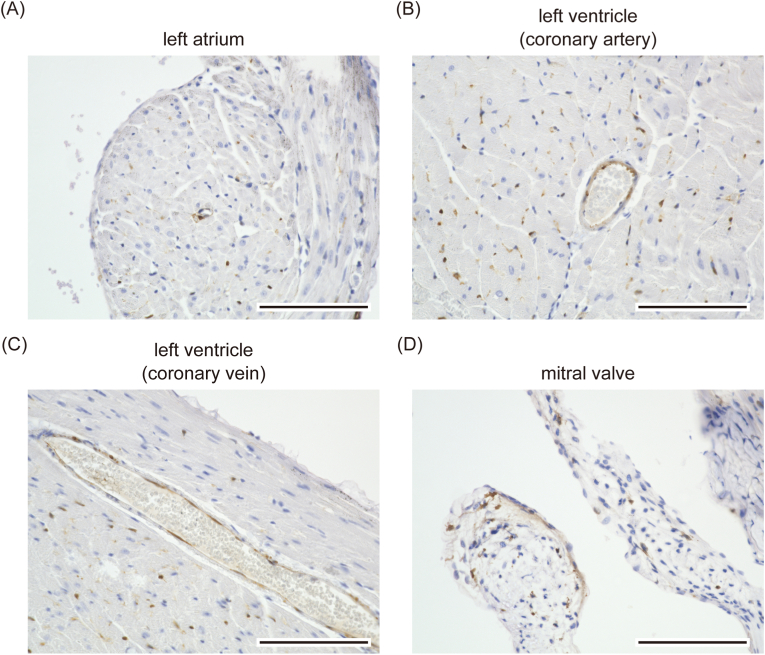
Immunohistochemistry for tDsRed marked past Notch1 signals Endothelial cells in the capillary (A-D), large artery (B), and vein (C) expressed tDsRed, suggesting Notch1 signals in their development. Some endocardial cells expressed tDsRed (D). Scale bars represent 100 μm.

**Fig. 4:**
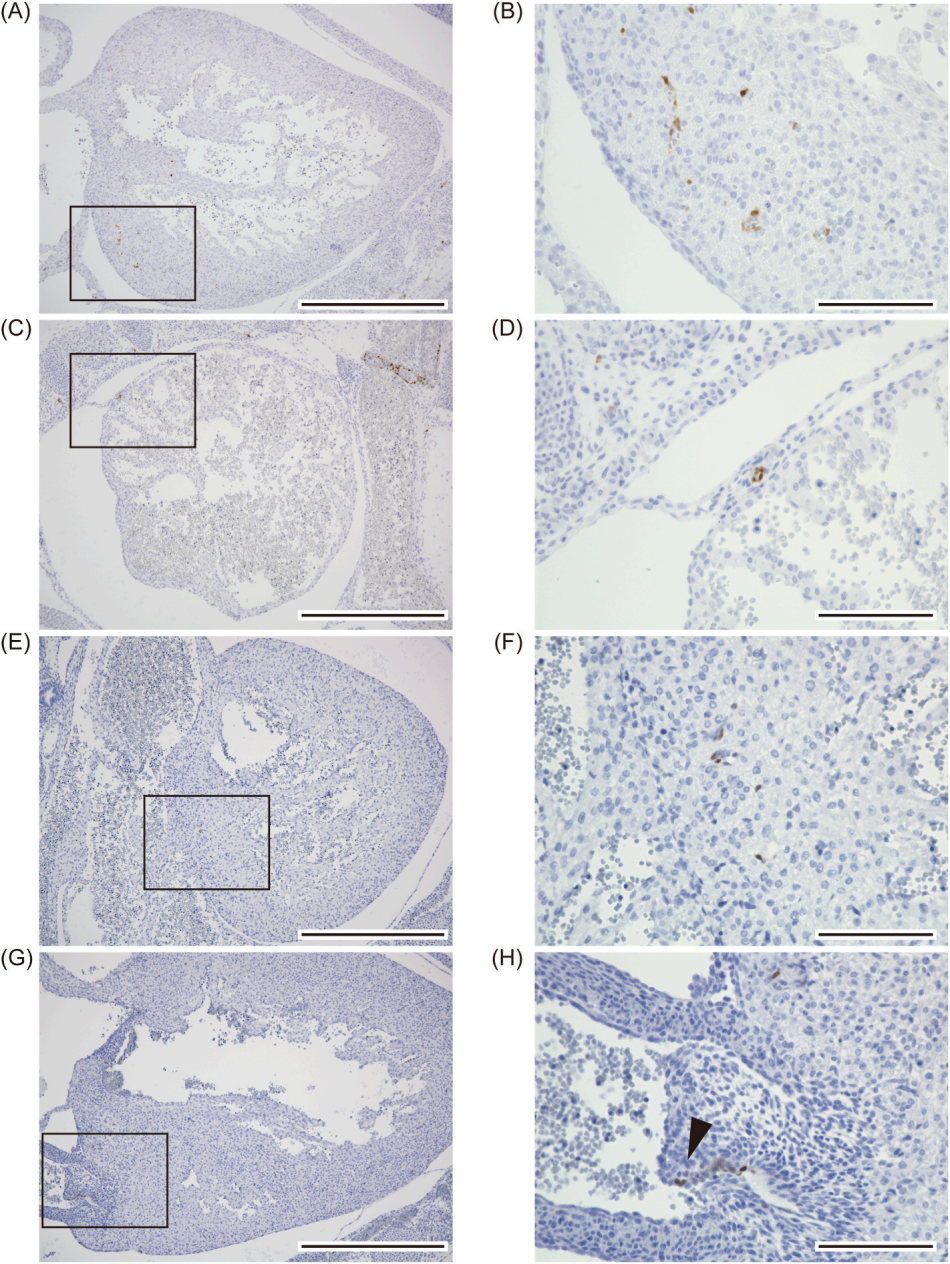
Immunohistochemistry for Cre recombinase marked ongoing Notch1 signals Cre recombinase was expressed in endothelial cells (A-F). One section showed Cre recombinase expression in the endothelial cells in the semilunar valve (G and H). Left panels (A, C, E, and G) are low magnification images, and right panels (B, D, F, and H) are high magnification images of the boxed areas in the left panels (A, C, E, and G). Scale bars represent 500 μm (A, C, E, and G) and 100 μm (B, D, F, and H).

### 3.3 Visualization of past Notch1 signal in the mouse heart

To examine Notch1 signal rather than Notch1 receptor expression, we conducted transgenic reporter mouse experiments. Briefly, binding of Notch1 receptor and its endogenous ligands allows cleavage of Gal4VP16 from Notch1-Gal4VP16 fusion receptor [27] and induces Cre recombinase expression [28]. Since Cre permanently labels the cell with tDsRed when combined with the R26GRR mouse [29], Cre and tDsRed represent ongoing and past Notch1 signals, respectively. Using this reporter system (Notch1-Gal4VP16; UAS-Cre; R26GRR mice), we examined tDsRed expression (past Notch1 signals) in adult mouse hearts (Fig. 3). We observed tDsRed expression in coronary endothelial cells, including large arteries (Fig. 3B) and veins (Fig. 3C), and a part of endocardial cells (mitral valve in Fig. 3D), consistent with re-analyses of human and mouse scRNAseq data. Although tDsRed expression in the cells lining the inner surfaces of vascular lumens suggested tDsRed expression in the endothelial cells, we examined a pan-endothelial marker (Pecam1) expression in these cells to further validate the identity of tDsRed-expressing cells (Supplementary Material 14). We observed colocalization of tDsRed and Pecam1 (arrowheads in Supplementary Material 14A, 14B, and 14D). In addition, a tDsRed-expressing cell was neighboring an EGFP-expressing thick cardiomyocyte, consistent with the notion that tDsRed-expressing cells were endothelial cells (arrows in Supplementary Material 14B, 14C, and 14E). These results suggest that endothelial cells received Notch1 signals during their development.

### 3.4 Visualization of the ongoing Notch1 signal in the mouse embryonic heart

Finally, we sought direct evidence of Notch1 signaling in the developing heart. We determined to examine the E14.5 heart since our scRNAseq re-analysis of mouse embryonic hearts suggested Notch1 expression in E14.5 (Fig. 2A and E), and this time point matches the rapid growth of compact working myocardium [32] that would require active sprouting angiogenesis for effective oxygen supply. Immunohistochemistry for Cre recombinase labeled surface-covering cells of the coronary vessels mostly in the outer (compact) layer of the developing heart of Notch1-Gal4VP16; UAS-Cre mice at E14.5 (the upper three panels in Fig. 4). One section also suggested Notch1 signaling in the surface cells of a semilunar valve (arrowhead in Fig. 4), suggesting a link between Notch1 signaling and outflow valve [4] and outflow tract formation [5]. In contrast to the embryonic stage, we did not observe Cre expression in an adult heart (Supplementary Material 15). In addition, although we observed spindle-shaped fluorescence from Cre-AlexaPlus488 and Cd31-Alexa647, we concluded that uncertainty remains regarding their colocalization, as background autofluorescence from blood cells was as high as the fluorescence from Cre-AlexaPlus488 and Cd31-Alexa647 (Supplementary Material 16). In summary, our findings morphologically support that endothelial cells receive Notch1 signaling during heart development, although the molecular signature of cells with ongoing Notch1 signaling remains unclear.

## 4 Discussion

Notch signaling is essential for heart development, although its mechanism remains unclear owing to the requirement for ligand binding and the high cellular heterogeneity in the heart. In this study, we combined scRNAseq re-analyses and a transgenic reporter assay that enabled Notch1 signaling visualization to show that coronary endothelial cells and a subset of endocardial cells receive Notch1 signals during development. Although cardiomyocytes were unlikely to receive Notch1 signals despite the presence of Notch1-associated LVNC and DORV, this remains plausible, as endothelial cells facilitate cardiomyocyte development and maturation via angiocrine signaling [33].

The scRNAseq samples we re-analyzed in this study were at mid-to late stages of heart development. The earliest time point in human samples (PCW6) roughly corresponds to mouse E14, when the secondary atrial septum appears, the left and right atrioventricular connections become separated, the proximal outflow tract becomes separated, and the semilunar valves develop [34]. Even earlier than this stage, the mouse heart at E10.5 already has atrioventricular cushions [34]. Therefore, we were unable to examine the very first stage (human PCW2 or mouse E7) of heart development, when the cardiac crescent forms [34]. Nevertheless, we believe that examination of mid-to late stages is reasonable when considering coronary vessel establishment. At mouse E10.5, sinus venosus endocardial cells begin to invade cardiac free walls to form the first coronary vascular population (the first wave) [15]. Approximately at mouse E11.5 (the second wave) and E18.5 (the third wave), endocardial cells of the ventricle begin to contribute to the coronary vasculature as the second coronary vasculature population, supporting the compartmentalization model of coronary vessel development [15]. Indeed, we observed confined Fabp4 (marker for coronary origin) expression (Fig. 2G) and a bit more widespread Nfatc1 (marker for sinus venosus endocardial origin) expression (Fig. 2H). Since Fabp4 is expressed in the endothelial cells on the epicardial side at mouse E15.5 [35], Cre recombinase-expressing endothelial cells (Notch1 signal receivers) at E14.5 might belong to Fabp4-expressing endothelial cells. Importantly, one section showed Cre recombinase expression (Notch1 signal) in semilunar valve endocardial cells, suggesting that the Notch1 signal acts across multiple endothelial cell lineages. To conclude the pan-endothelial cell lineage action of Notch1, however, co-labeling of Cre recombinase (Notch1 signal) and cell lineage markers is necessary, which is beyond the scope of this study.

It might seem ambiguous that we observed expressions of Cre recombinase (ongoing Notch1 signal) and tDsRed (past Notch1 signal) in a limited part of the endocardium, although this does not impede the importance of Notch1 signaling in this tissue. In addition to the inevitable imperfection of transgenic technologies, there is strong evidence of Notch1 signals in the endocardium during ventricular maturation and compaction [36,37]. Nevertheless, a clear description of ongoing and past Notch1 signaling in surface-covering cells in the heart and a subset of endocardial cells would facilitate our understanding of this signaling in normal heart development and, thus, the pathogenesis of congenital heart diseases.

Finally, endothelial Notch1 has been linked to proper development of the semilunar valves and outflow tract, as Tie2-Cre-mediated endothelial cell-specific haploinsufficiency of Notch1 increased the incidence of thickened aortic or pulmonary valves in a Nos3-null background [38]. In addition, one paper suggested that Tie2-Cre-mediated overexpression of the Notch1 intracellular domain promoted endothelial-to-mesenchymal transition in the developing heart, which is involved in semilunar valve development, via increased Snail expression [39]. These reports suggest Notch1 activity in endothelial cells, including endocardial cells, during heart development, although its downstream target genes and role in coronary development remain unclear. This study morphologically suggested Notch1 signaling in endothelial cells by directly labeling the past (Fig. 3) and ongoing (Fig. 4) Notch1 signals, although further characterization of the molecular signature of the signaling-receiving cells should be necessary. Since our study is observational, how Notch1 signal supports heart development, including the downstream target genes of this signal, is a scope of future studies.

## 5 Conclusion

Coronary endothelial cells and a subset of endocardial cells were suggested to receive Notch1 signals. This finding and future detailed analysis of spatiotemporal Notch1 signals would facilitate understanding of normal and abnormal heart development.

## CRediT authorship contribution statement

**Yoshitoku Watabe:** Data curation, Formal analysis, Investigation, Software, Visualization, Writing – original draft. **Satoru Takahashi:** Supervision. **Masaharu Yoshihara:** Conceptualization, Formal analysis, Funding acquisition, Investigation, Methodology, Project administration, Software, Visualization, Writing – original draft.

## Ethics declaration

This study was conducted in accordance with the following guidelines for animal welfare and/or reporting: The institutional or national guidelines of the University of Tsukuba and Japan. This study was approved by the University of Tsukuba (Approval No. 22–059, 23-049, 24-133, 25-150, 26-305 (Approval Numbers for Animal Experiments) and 220018 (Approval Number for DNA Experiments)).

## Declaration of competing interest

The authors declare the following financial interests/personal relationships which may be considered as potential competing interests:Masaharu Yoshihara reports financial support was provided by Japan Society for the Promotion of Science. If there are other authors, they declare that they have no known competing financial interests or personal relationships that could have appeared to influence the work reported in this paper.

## Figures and Tables

**figs1:**
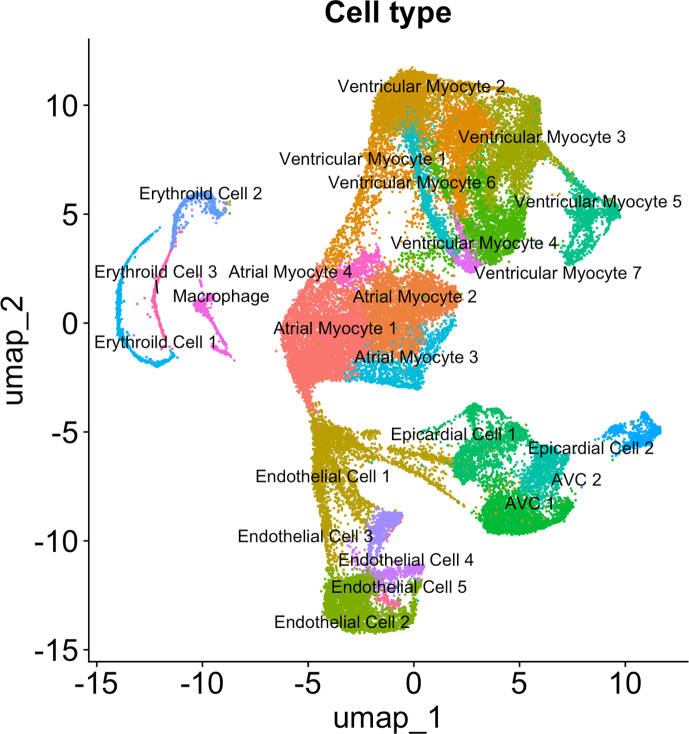


**figs2:**
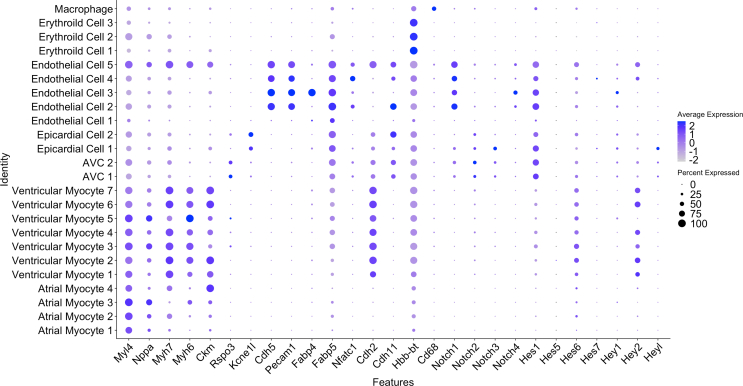


**figs3:**
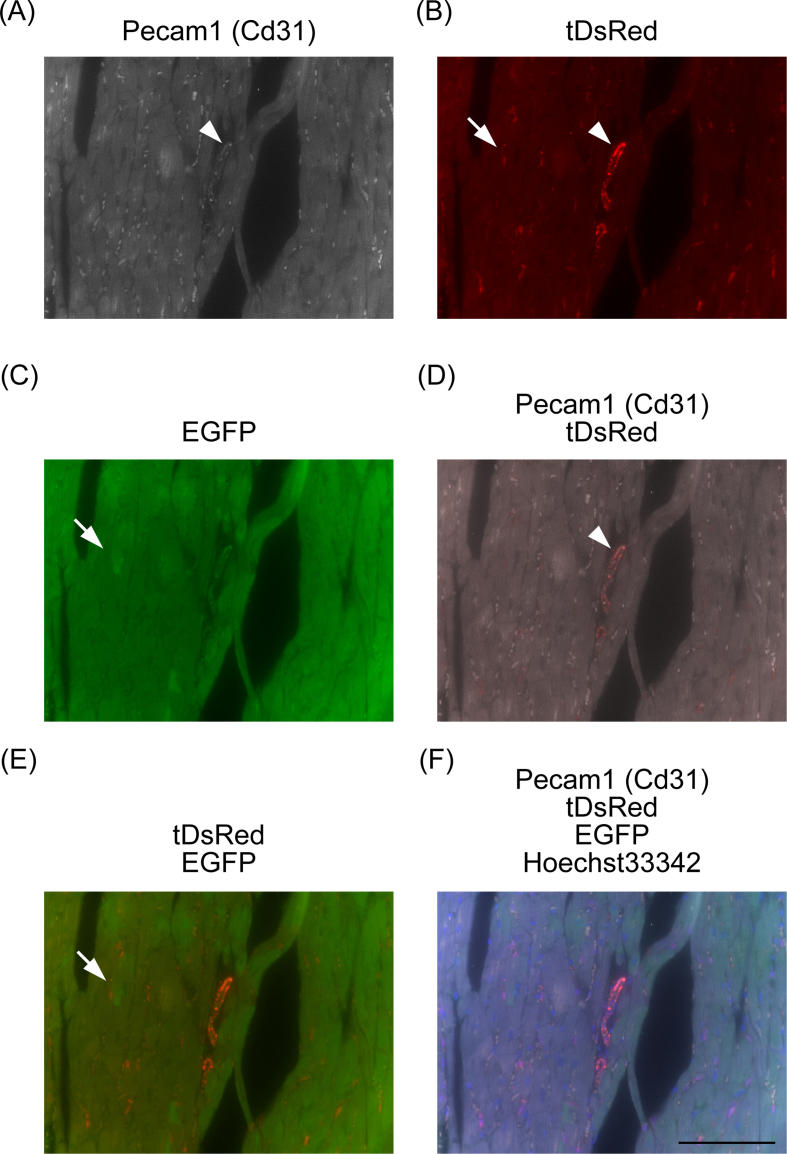


**figs4:**
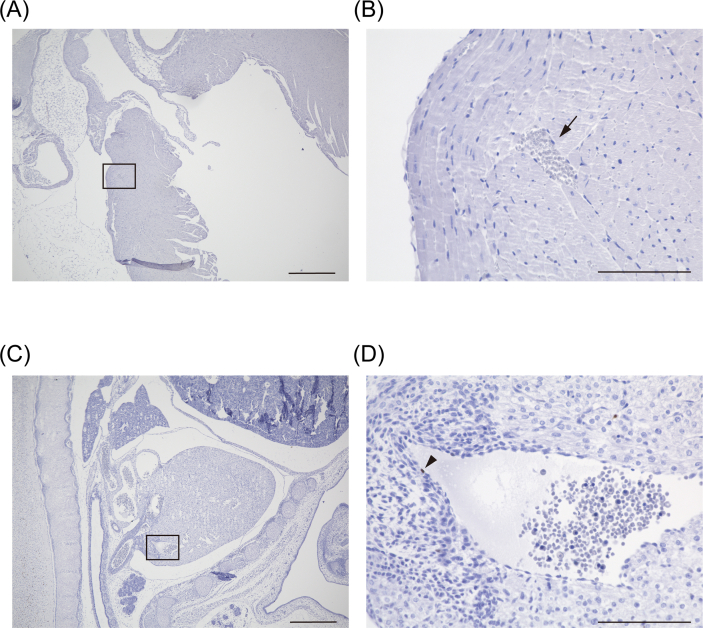


**figs5:**
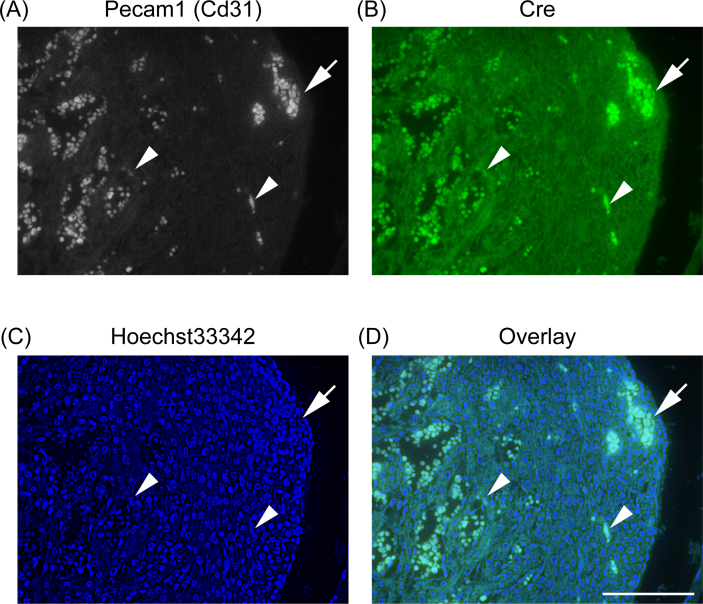


## Data Availability

The R code used in the present study is available in our archived repository (https://zenodo.org/records/19884702) under the terms of the Creative Commons Zero v1.0 Universal.
